# Design and evaluate the performance of a mechanical system for the release of *Harmonia axyridis* adults

**DOI:** 10.3389/fpls.2024.1297182

**Published:** 2024-03-05

**Authors:** Xiao-Ya Dong, Xiang Tong, Jing Ma, Bai-Jing Qiu

**Affiliations:** ^1^ Key Laboratory of Modern Agricultural Equipment and Technology, Ministry of Education, Jiangsu University, Zhenjiang, Jiangsu, China; ^2^ Key Laboratory of Plant Protection Engineering, Ministry of Agriculture and Rural Affairs, Jiangsu University, Zhenjiang, Jiangsu, China

**Keywords:** biological control, mechanical release, *Harmonia axyridis*, natural enemies, mechanical distribution

## Abstract

*Harmonia axyridis* (*H. axyridis*) is the natural enemy of many aphid species. Traditional manual release of *H. axyridis* adults requires substantial manpower, and release efficiency is low. Automatic mechanical devices can improve the efficiency of delivery. Based on *H. axyridis* adults’ morphological size, a prototype release system for *H. axyridis* was designed, which considered the adhesion characteristics of *H. axyridis* adults. According to the measured physical characteristics of *H. axyridis* adults, the structural parameters of the mechanical system for the release of the *H. axyridis* adults were determined. The relationship of the quantity of release, the impeller rotating speed, and the time for the release of *H. axyridis* adults were constructed. The mechanism can quantitatively adjust the number of *H. axyridis* adults to meet a certain *H. axyridis*–aphids ratio. Combining the image processing technology with the camera function of a mobile phone, the maximum cross-sectional area method was used to count the *H. axyridis* adults in the designated area. Results showed that the impeller rotating speed had a significant effect on the survival rate of the *H. axyridis* adults. When the airflow velocities were 29.5 m/s and 38.3 m/s, the survival rates of the *H. axyridis* adults were 93.8% and 94.5% at 4.2 rpm. The adhesion rate of the *H. axyridis* adults was 2.5%–4.6%. This work will provide technical support for the research of biological control.

## 1 Introduction

The primary method for protecting crops from pests is currently chemical control. However, the long-term use of chemical insecticides can lead to pest resistance and compromise the effectiveness of pest control (Alyokhin et al., 2007; Andrei et al., 2008; Yu and Powles, 2014). Biological control is a sustainable and environmentally friendly approach (Pilkington et al., 2010). This approach usually uses natural enemies to manage pests such as insects, mites, weeds, and plant diseases (Lanzoni et al., 2017). Compared to chemical control, biological control has been widely recognized as a promising solution to address environmental pollution and pesticide residues (Gan-Mor and Matthews, 2003). Therefore, biological control has attracted considerable interest in the field of crop protection. However, the manual release of natural enemies for crop protection necessitates a large labor force. The workers who released natural enemies were required to endure a prolonged period in a humid and hot environment, experiencing fatigue and discomfort. To solve this issue, considerable efforts have been dedicated to creating mechanical systems for the distribution of natural predators.

Since the advent of mechanical systems for the release of natural enemies (Pickett et al., 1987), numerous strategies have been developed to cater to varying release requirements. Based on the type of external force employed, these mechanical systems for releasing natural enemies can be categorized into three classes. The first class is the centrifugal release system, which utilizes centrifugal force generated by the rotation of a disc or sliding plate to eject predators (Giles et al., 1995; Casey and Parrella, 2005; Blandini et al., 2008; Emma et al., 2010; Zappala et al., 2012). In a typical centrifugal release system, predators are enclosed in a hopper with carrier materials, and the release process is powered by two electric motors. The second class is the pneumatic release system. The natural predators were released into a diffuser by force of gravity (Papa et al., 2018). The diffuser relies on a centrifugal fan to generate high-speed airflow to transport the natural predators. Pezzi and his coworkers (Pezzi et al., 2015) designed a novel air-assisted release prototype, wherein a commercial bottle filled with beneficial organisms and substrate can be inverted on the extraction system. The extraction system is constituted of a push rod with a metal spike with reciprocating movement generated by an electromagnet powered by a handheld battery. Last, the beneficial arthropods were transported via airflow. The last class is the vibration release system. The vibration release system combines vibration force with gravity. The predator-releasing device is fixed to the tractor using a three-point suspension in this system. As the tractor moves, Perillus binoculars are transported to the designated location. Using this release system, Khelifi and his work team (Khelifi et al., 2011) deployed two spotted stink bugs to manage the Colorado potato beetle. The aforementioned mechanical systems for the release of natural enemies predominantly release predators with a volume at the micron level. These systems disseminate mixtures of mites and carrier materials (Giles et al., 1995; Opit et al., 2005).

*Harmonia axyridis* (*H. axyridis*) has been extensively introduced for the biological control of agricultural pests (Haelewaters et al., 2017). These agricultural pests include aphids, bugs, psyllids, moths, and others. Instar larvae often exhibit cannibalism behavior (Osawa, 1992), and adult *H. axyridis* are more proficient at preying on pests. Consequently, *H. axyridis* adults are frequently released in fields or greenhouses for biological control purposes. To date, the mechanicalization of *H. axyridis* adult release has not been significantly advanced. Based on previous research, combining the first type of centrifugal release system and the second type of pneumatic release system, we design a release device that combines the centrifugal and the centrifugal. Based on the morphological size, area-restricted searching behavior, and adhesion properties of *H. axyridis* adults, we have developed a prototype for the release of *H. axyridis* adults. The prototype can be carried over the shoulder. The prototype can perform the following functions: (i) continuous or cycle-interrupted release and (ii) adjustment of working parameters: distribution distance and the number of *H. axyridis* adults. To assess the distribution performance of the prototype, the survival rate, distribution uniformity, and adhesion rate of *H. axyridis* adults have been characterized.

## 2 Materials and methods

### 2.1 Morphological size of *H. axyridis* adults

**Figure 1:**
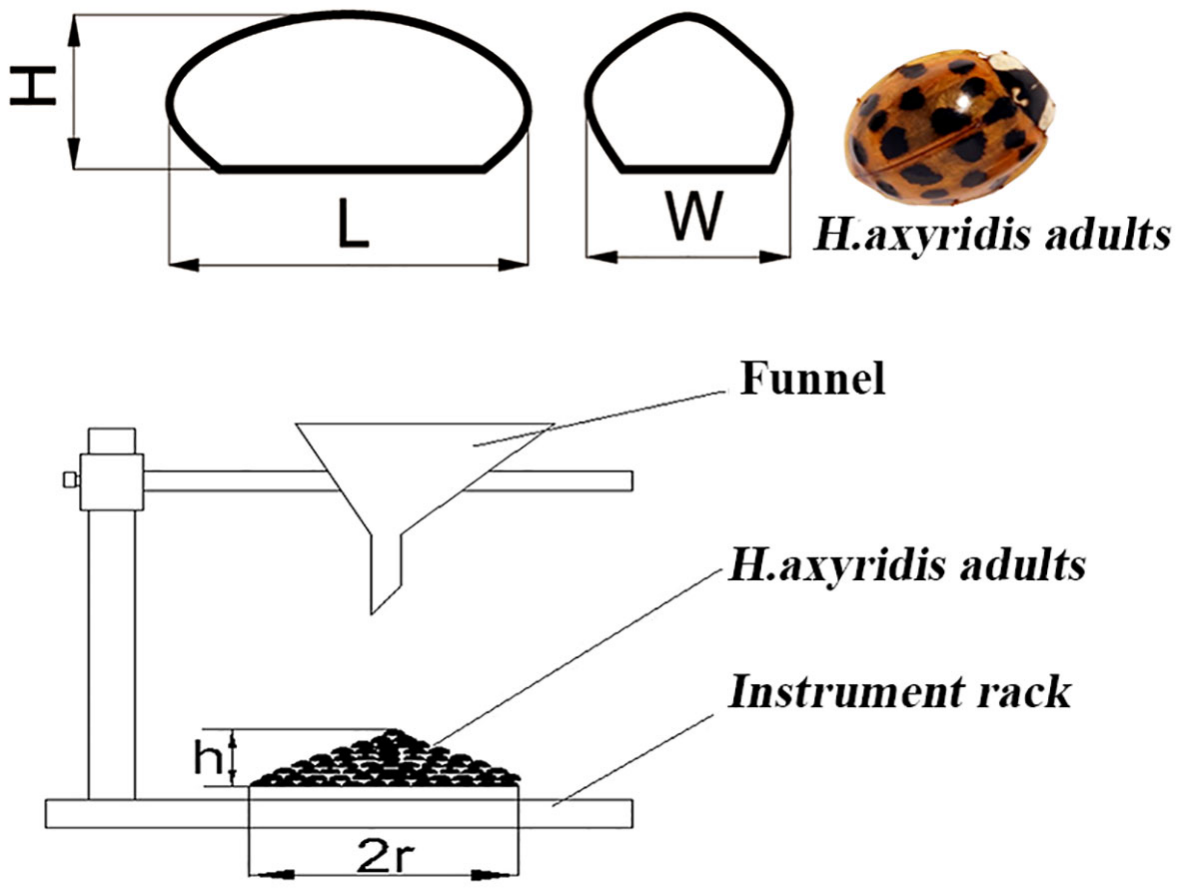
Measurement schematic of *H. axyridis* adults morphological size. L, W, and H are the length, width, and height of *H. axyridis* adults. h and 2r are the height and bottom surface length of the cone.

The morphological size of adult *H. axyridis* individuals is a crucial parameter in the design process of mechanical systems for the release. Therefore, *H. axyridis* adults purchased from a commercial insectary (J.D. Crop Protection Market, China) were used in this study. To overcome the challenges posed by the active movements of *H. axyridis* adults, 50 samples were placed in airtight tubes for 30 min to lose activity. As shown in **Figure 1**, the *H. axyridis* adult was approximated as a semi-ellipse. The length (L) of an *H. axyridis* adult was determined by measuring the distance from its head to its tail. The width (W) was defined as the distance perpendicular to the length in the horizontal plane. The H shown in **Figure 1** is the height (H) of the *H. axyridis* adult. The L, W, and H of *H. axyridis* adults were measured using a digital display vernier caliper (German Knight MNT-150). The weight (m) of *H. axyridis* adults was determined using the electronic balance (Sartorius BT-125D).

To design an *H. axyridis* adult population adjustment mechanism, it was necessary to measure the angle of repose (φ) of *H. axyridis* adults. The φ has reference significance for the design of the tilting angle of the wall of the *H. axyridis* adults container (Amidon et al., 2017). Theoretically, the tilting angle of the wall of the *H. axyridis* adults container should be smaller than φ of the *H. axyridis* adults, which is conducive to the flow of *H. axyridis* adults. A sample of 50 g of *H. axyridis* adults was packed into a funnel and freely dropped onto an instrument rack. H. Axridis adults form a cone pile on the instrument rack. The accumulation height h and the maximum section length of the bottom surface 2r were measured, and the angle of repose was calculated using the equation: φ = arctg h/r. The angle of repose was repeated five times. The morphological parameters of *H. axyridis* adult individuals are listed in **Table 1**.

**Table 1:** The morphological parameters of *H. axyridis* adult individuals.

Characteristics	Unit	Value
Length (L)	mm	5.89 ± 1.21
Width (W)	mm	4.29 ± 1.46
Height (H)	mm	1.95 ± 0.87
Angle of repose (φ)	°	36.32 ± 5.17
weight (m)	mg	21.36 ± 4.24

### 2.2 Description of the mechanical system for the release of *H. axyridis* adults

The prototype of mechanical for release *H. axyridis* adults was designed to meet the following: (i) the *H. axyridis* adults are unharmed when they are released smoothly and (ii) can be carried on the shoulder of the operator.

**Figure 2:**
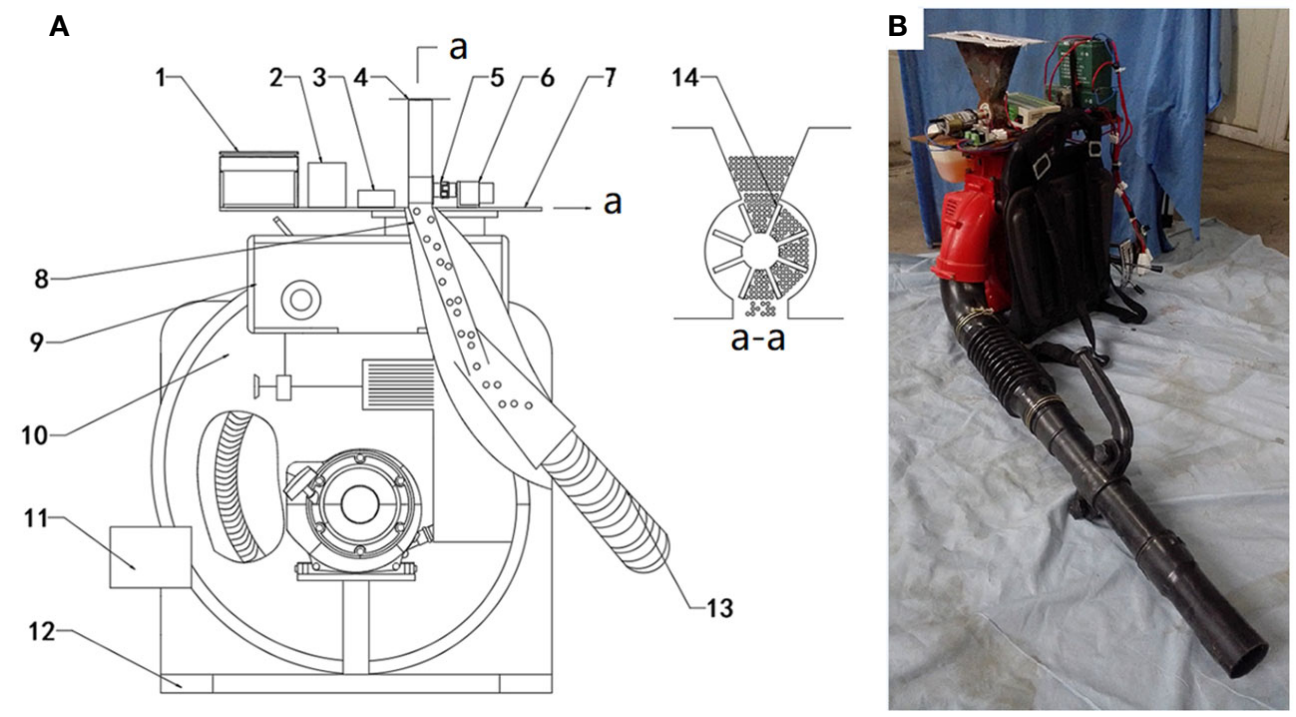
**(A)** Structure diagram of release system for *H. axyridis* adults: 1, lithium battery; 2, relay; 3, PLC module; 4, container; 5, coupling; 6, DC motor; 7, joint plate; 8, rotating plate; 9, petrol tank; 10, centrifugal fan; 11, control board; 12, frame; 13, air channel; 14, impeller. **(B)** Release system for the release of *Harmonia axyridis* adults.

To meet the requirement of mechanical release, we designed a release system for the distribution of *H. axyridis* adults. The structure of the release system is depicted in **Figure 2**. The release system mainly consisted of two parts. One was a mechanical section, and the other was a control section. The mechanical section included a centrifugal fan, rotating plate, impeller, and spray duct. The control section included a programmable logic controller (PLC) module, relay, and DC motor. The main characteristics of the prototype are detailed in **Table 2**.

**Table 2:** Main characteristics of the release system.

Characteristics	Unit	Value
Length	mm	590
Width	mm	320
Height	mm	600
Weight	kg	11.5
Capacity *H. axyridis* container	L	0.8
Matching power	kw	1.95
Air channel diameter	mm	75
Airflow velocity	m s^−1^	29.5–38.3
Maximum horizontal distribution distance	m	5
*H. axyridis* number adjustment range		60–300

The working principle is as follows: the gasoline engine drives the rotation of the centrifugal fan. The airflow velocity in the air channel is regulated by adjusting the speed of the centrifugal fan. The lithium battery supplies power to the PLC module and the DC motor. A timer in the PLC module regulates relay timings and determines how long the DC motor stays powered. During operation, the DC motor drives an adjustment mechanism to release *H. axyridis* adults, enabling them to enter the air channel. At high-speed airflow, *H. axyridis* adults are propelled through an air channel for their release. A 70° incline of the rotating plate relative to the horizontal plane is set for *H. axyridis* adults to effectively enter the air channel. If there is any malfunction in circuit control components, a drawbar is employed to rotate and align with the horizontal plane angle position. By manipulating the drawbar, the *H. axyridis* adults can be prevented from entering the air channel, effectively preventing further release. The mechanical system for the release of *H. axyridis* adults can be carried on the back via two shoulder straps.

### 2.3 Adjustment mechanism for the release of *H. axyridis* adults

**Figure 3:**
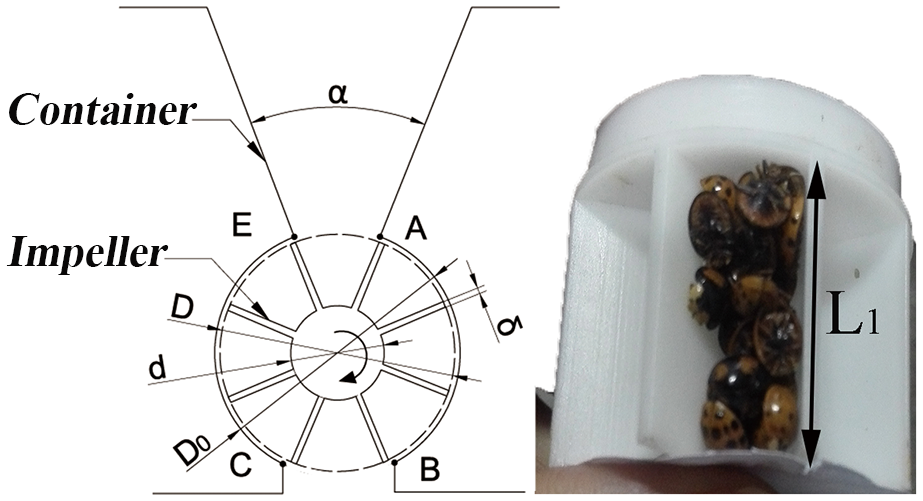
*H. axyridis*–transport mechanism.

The crucial component of the mechanical system facilitating the release of *H. axyridis* adults was the adjustment mechanism. It included a coupling, a DC motor, an impeller, and a container. The *H. axyridis*–transport mechanism is illustrated in **Figure 3**. Because of gravity, the *H. axyridis* adults moved toward the space between the vanes. Upon rotation of the DC motor driving the impeller, *H. axyridis* adults were funneled toward the BC exit and cascaded into the air channel. The release of *H. axyridis* adults was completed with the addition of high-speed airflow. To ensure a uniform and steady release of *H. axyridis* adults, eight vanes were designed at equal intervals along the periphery of the impeller. The angle between two adjacent vanes was labeled as α, and the angle of repose was denoted as should correspond. Based on the value of the angle of repose measured in **Table 1**, we determined that the value of α is 45°.

For normal operation of the impeller, a gap between the impeller and the container’s shell was necessary. Otherwise, a malfunction may occur during the coordinated movement between the shell and the impeller. The radial gap between the impeller and the shell should satisfy the following conditions:


(1) $$0<\frac{{\text{D}}_{0}-\text{D}}{2}\;<\{\text{L},\text{ W},\text{H}\}$$

Here, *D*_0_ represents the diameter of the container’s shell, *D* denotes the external diameter of the impeller, and L, W, and H correspond to the length, width, and height of *H. axyridis* adults, as visualized in **Figure 2**.

To ensure the proper distribution of *H. axyridis* adults between the vanes and a smooth drop from the BC exit, the length of line segment BC should slightly exceed the maximum distance between two adjacent vanes.


(2) $$\text{ BC}>\text{D}\cdot \sin\left(\frac{\text{α}}{2}\right)\;$$

Assuming that *H. axyridis* adults have a semi-ellipsoid shape, we calculated the average volume of each individual adult:


(3) $$V_{1}=\frac{1}{2}\times \frac{4}{3}\text{π}\cdot \left(\text{L}\cdot \text{W}\cdot \text{H}\right)$$

The total volume of the space between the vanes that can accommodate *H. axyridis* adult is


(4) $$\text{V}=\left[\text{π}\cdot \frac{\left({\text{D}}^{2}-{\text{d}}^{2}\right)}{4}-\text{n}\cdot \text{δ}\cdot \frac{\text{D}-\text{d}}{2}\right]\cdot L_{1}$$

Here, *d* represents the impeller’s inner diameter,  δ represents the thickness of a vane, $L_{1}$ represents the length of the impeller. From **Equations 2**, 3, it can be deduced that a single space can house a certain number of *H. axyridis* adults.


(5) $$\text{N}=\frac{V}{\text{n}\cdot V_{1}}$$

Linking Points A and C, Points B and E in line, both line AC and line BE pass through the center of the circle. The initial position is shown in **Figure 3**. The time it takes for *H. axyridis* adults to fall into an air channel from the container is


(6) $$\text{T}=\frac{37.5}{{\text{n}}_{1}}+\left[\frac{\;}{\text{SN}}\right]\cdot \frac{7.5}{{\text{n}}_{1}}$$

where *n*_1_ represents the impeller rotating speed, *S* represents the total number of *H. axyridis* adults in the container, and [] is the round symbol.

### 2.4 Test of *H. axyridis*–transport mechanism

To test the validity of Equation 6, an experiment was carried out. Initially, a specific quantity(S)of an *H. axyridis* adult was poured into a container. The value of S was predetermined as 60, 120, 180, 240, or 300. A reflective paper was attached to the coupling, and the tachometer tracked the coupling’s real-time speed. Then, subsequently, the impeller rotating speed was adjusted. Since the coupling is connected to the impeller, the rotation speed of the coupling is equal to that of the impeller. The tachometer recorded impeller rotating speeds (n1). The release time (t) of *H. axyridis* adults was recorded. The experiment was replicated twice.

### 2.5 Statistical analysis

#### 2.5.1 Camera calibration and counting of *H. axyridis* adults

To investigate the distribution, it was necessary to count the number of *H. axyridis* adults in designated areas. Traditional counting methods include weighing and manual counting (Opit et al., 2005; Pezzi et al., 2015). However, these approaches are time consuming and challenging due to the mobility of *H. axyridis* adults.

**Figure 4:**
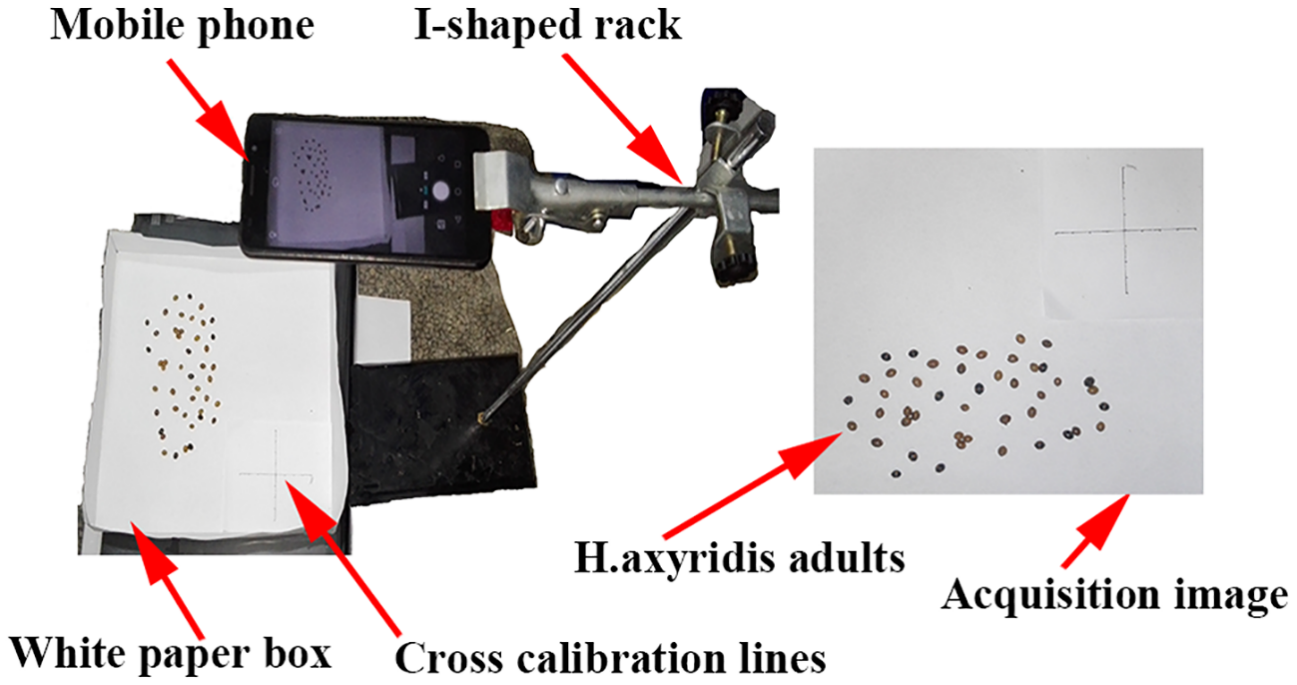
Camera calibration device and acquisition image.

For improved counting efficiency, an image processing method based on MATLAB (2012a) software was developed (refer to the Supporting Information for further details on the procedure code). When counting *H. axyridis* adults in a particular region, there is often a problem with *H. axyridis* adult adhesion. If we do not segment the attached *H. axyridis* adults, the accuracy of the final count will be affected. We used the watershed algorithm, the corrosion algorithm, and the maximum cross-sectional area method at the beginning of the experiment. We then found that the maximum cross-sectional area method was the most accurate for counting adult *H. axyridis* adults. Therefore, the method was chosen to count *H. axyridis* adults in the following experiment. When reading the image using MATLAB, each pixel represents the area of an *H. axyridis* adult in the picture. Considering ease of use, a phone’s built-in camera (H60-L01, Huawei, Resolution: 1920×1080 pixels) was selected as the hardware for capturing images. However, factors such as camera angle and distortion can impact image accuracy. Consequently, a camera calibration experiment was conducted, as illustrated in **Figure 4**.

**Figure 5:**
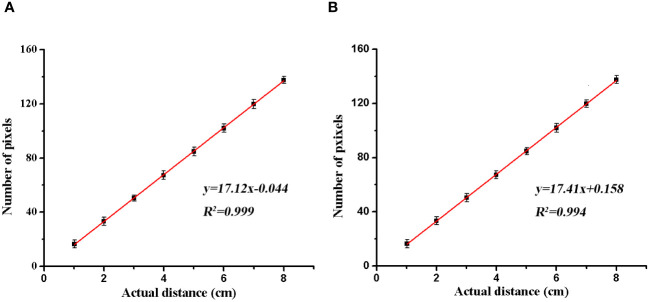
The fitting graph of the pixels to the actual distance. **(A)** The horizontal direction; **(B)** the vertical direction.

A white paper box (25 cm × 25 cm × 5 cm) was placed horizontally. A retort stand and clamp were used to keep the mobile phone parallel to the white paper box (**Figure 4**). The vertical distance from the lens to the center of the white box is 45 cm. This distance ensures that the phone’s built-in camera fully captures the white paper box. A cross-calibration line on white paper (8 cm × 8 cm) was employed to calibrate the true value of the pixel. Each cross-calibration line was divided into eight 1-cm segments. The least squares method had been utilized to fit the pixels to the actual distance. The fitting graph is shown in **Figure 5**. **Figure 5A** is the fitting graph of the actual distance to the number of pixels in the horizontal direction, and **Figure 5B** is that of the vertical direction.

#### 2.5.2 Performance experiment of the mechanical distribution prototype

**Figure 6:**
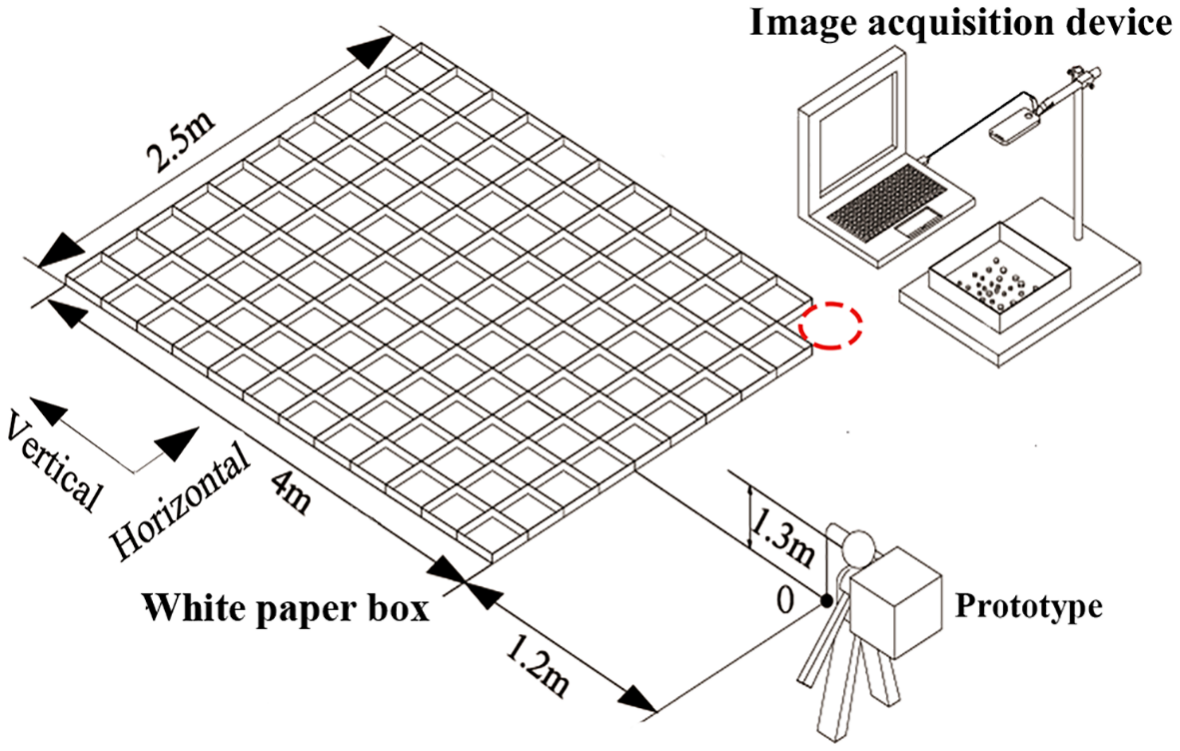
Schematic diagram of the release experiment.

To evaluate the survival, uniformity of distribution, and adhesion rate of *H. axyridis* adults after release, the experimental design is shown in **Figure 6**. A 4.0 m × 2.5 m rectangle was established using interconnected white paper boxes. To prevent the white paper boxes from being blown away by the wind, use thumbtacks to secure them to the ground. After carefully pouring a specific quantity of *H. axyridis* adults into the container, the operator stood motionless at the release point o with the release device fastened to his back (as shown in **Figure 6**). To ensure that the *H. axyridis* adults fell into the rectangle, the release point was positioned 1.2 m away from the rectangle formed by the connected white paper boxes. This distance was established through a preliminary experiment. The air channel tube was positioned parallel to the ground at an outlet height of 1.3 m. Next, the power supply was initiated, and the operator sets the impeller rotation speed and airflow velocity. The *H. axyridis* adults were then released by activating the switch. After the release of the *H. axyridis* adults, the machine was turned off. The operator remained stationary throughout the process. The natural wind velocity during the experiment ranged from 0.2 to 0.4 m/s, and the ambient temperature was 23.4°C. The effects of different impeller rotating speeds (4.2 rpm, 9.8 rpm, and 16.8 rpm) and airflow velocities (29.5 m/s and 38.3 m/s) on the distribution, survival, and adhesion of *H. axyridis* adults were investigated.

Taking point O as the origin in **Figure 6**, a coordinate system was established. The horizontal direction was perpendicular to the air channel, and the vertical direction was parallel to the air channel. The number of *H. axyridis* adults in the paper boxes was counted using an image processing method based on MATLAB (2012a, MathWorks Inc.) software (see Supporting Information for further details of the procedure code). The distribution of the *H. axyridis* adults was calculated. The number of dead *H. axyridis* adults in every box and the number of *H. axyridis* adults attached to the walls of the container were recorded.

The coefficient of variation (CV) of horizontal or vertical distribution was defined as Equation 7:


(7) $$CV=\frac{SD}{Mean}\times 100$$

Where SD is the standard deviation of the number of *H. axyridis* adults in the white paper boxes of each row or column, Mean represents the average *H. axyridis* adults number of in each row or column of the white paper boxes.

In the process of distribution, some *H. axyridis* adults adhered to the container wall, resulting in incomplete release. The adhesive force of *H. axyridis* adults amounts to approximately five times their body weight (Ishii, 1987). Adhesion rate (AR) was calculated according to Equation 8.


(8) $$\text{AR}=\frac{{\text{N}}_{1}}{\text{T}+{\text{N}}_{1}}\times 100\%$$

Where N_1_ represents the number of *H. axyridis* adults adhering to the container wall and T represents the total number of *H. axyridis* adults in the white paper boxes.

During the release, *H. axyridis* adults came into contact with the impeller and were distributed by the airflow in the air channel. After distribution, the survival rate (SV) was evaluated using Equation 9.


(9) $$\text{SV}=\left(1-\frac{{\text{N}}_{2}}{\text{T}}\right)\times 100\%$$

Where N_2_ represents the number of deceased *H. axyridis* adults.

## 3 Results and discussion

### 3.1 Quantitative adjustment model

The number of *H. axyridis* adults accommodated by the space between the vanes is an important factor in determining the parameter of impeller rotating speed. If the release amount of *H. axyridis* adults is certain, the space between the vanes contains more *H. axyridis* adults, and then the impeller rotating speed can be relatively reduced. According to the morphological size of the *H. axyridis* adults as shown in **Table 1** and the Equations 1–5, the other parameters of the impeller (**Figure 3**) were determined as follows: *D* = 40 mm, *d* = 15 mm, δ = 2 mm, *L*_1 =_ 30 mm, *D*_0 =_ 43 mm, BC = 18 mm. Due to manufacturing errors, actual values are *D* = 40.72 mm, *d* = 14.84 mm, δ = 1.77 mm, *L*_1 =_ 30.57 mm, *D*_0 =_ 43.08 mm, BC = 18.63 mm. The materials for the impeller are ABS plastic and the container is no. 45 steel. Considering the complex factors such as the impeller rotating speed (0–24 rpm) and the ratio of *H. axyridis* adults to aphids, the quantity of *H. axyridis* accommodated in the space between the vanes (N) was established as 35.

To determine if the actual number of *H. axyridis* adults found in the space between the vanes was consistent with the theoretical value, a certain amount of *H. axyridis* adults were introduced into the space and the experiment was repeated ten times. The resulting number of *H. axyridis* adults was recorded and analyzed. The results showed that there were 33 ± 3.06 *H. axyridis* adults identified, with an error rate of 5.7% compared to the predetermined value. The errors were mainly caused by three factors: (i) the morphology of *H. axyridis* adults was similar to that of a semi-ellipsoid, but not a normal semi-ellipsoid. When the *H. axyridis* adults were in contact with the impeller vanes, there was a seam between the *H. axyridis* adults and the container wall, (ii) errors in processing techniques, and (iii) individual differences in the size of *H. axyridis* adults.

To evaluate the quantitative release performance of the mechanical system for *H. axyridis* adults, we can not only control the number of *H. axyridis* adults to test the release time but also control the number of *H. axyridis* adults to test the release time. Considering the convenience of the experiment operation, this experiment adopts the number of *H. axyridis* adults controlled to test the release time. To determine the relationship between theoretical and actual values of release time, a serials of experiments was implemented. The release time is shown in **Table 3**. Setting the needing time t as the dependent variable, the total number of *H. axyridis* adults S and the impeller rotating speed n_1_ as an independent variable. Linear regression was performed using the SPSS 17.0. The fitting equation was established as Equation 10:

**Table 3:** Experimental data of *H. axyridis* adults release time.

Impeller rotating speed (rpm)	Number of *H. axyridis* adults				
	60	120	180	240	300
4.2	11.95 ± 0.51	14.71 ± 0.03	19.00 ± 0.68	22.84 ± 0.57	28.45 ± 0.83
7.4	7.24 ± 0.29	9.21 ± 0.03	11.33 ± 0.02	13.48 ± 0.09	15.90 ± 0.32
9.8	5.10 ± 0.35	7.36 ± 0.81	8.38 ± 0.05	9.78 ± 0.19	11.54 ± 0.13
14.3	4.13 ± 0.21	5.44 ± 0.02	6.38 ± 0.06	7.42 ± 0.01	8.85 ± 0.16
16.8	3.87 ± 0.08	4.72 ± 0.01	5.32 ± 0.15	5.66 ± 0.33	7.18 ± 0.23
21.2	3.18 ± 0.08	4.09 ± 0.06	4.93 ± 0.13	4.95 ± 0.59	5.94 ± 0.10
24	2.77 ± 0.13	3.41 ± 0.35	4.32 ± 0.15	4.04 ± 0.13	5.26 ± 0.30


(10) $$\text{t}=0.808+27.897\times \frac{1}{{\text{n}}_{1}}+0.277\times \frac{\text{S}}{{\text{n}}_{1}}$$

The correlation coefficient of R^2^ is 0.943, which means the fitting is well.

**Figure 7:**
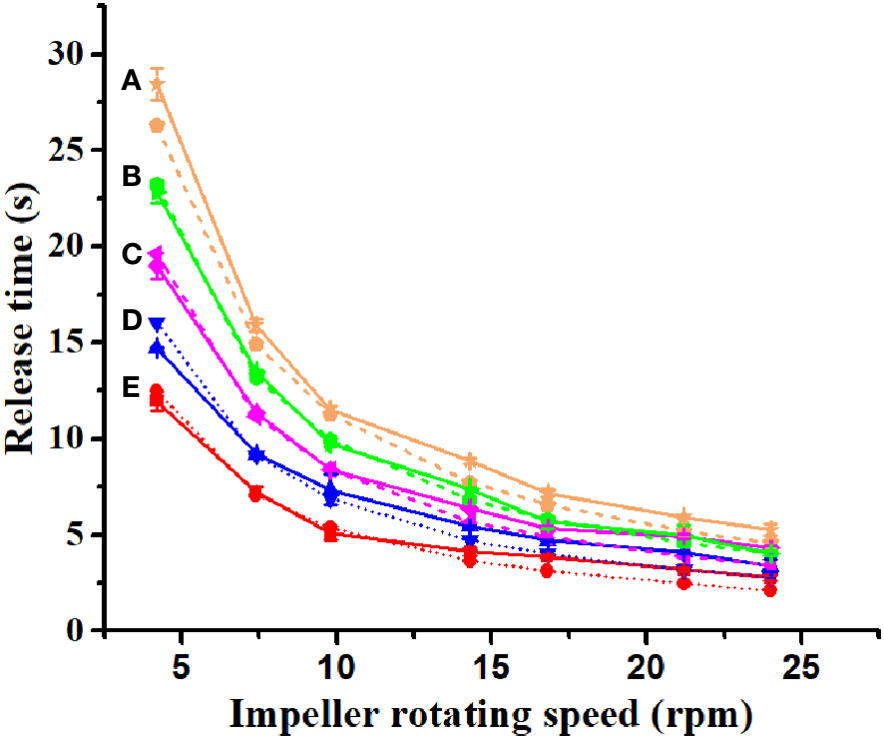
The theoretical (dotted line) and experimental values (solid line) of the time for the release of *H. axyridis* adults at different impeller rotating speeds. The number of *H. axyridis* adults was 300, 240, 180, 120, and 60 (A–E).

To evaluate the precision of the release mechanism, different quantities of *H. axyridis* adults were released via the release system. The obtained results were compared to the theoretical results, as shown in **Figure 7**. The dotted line is the theoretical value of the release time under different impeller rotating speeds. The solid line is the value measured by the experiment. As shown in **Figure 6**, there is a consistent correlation between the theoretical and actual values, indicating a good agreement between the two. The result shows that controlled release can be achieved by adjusting the impeller rotating speed.

### 3.2 Counting based on image processing

**Figure 8:**
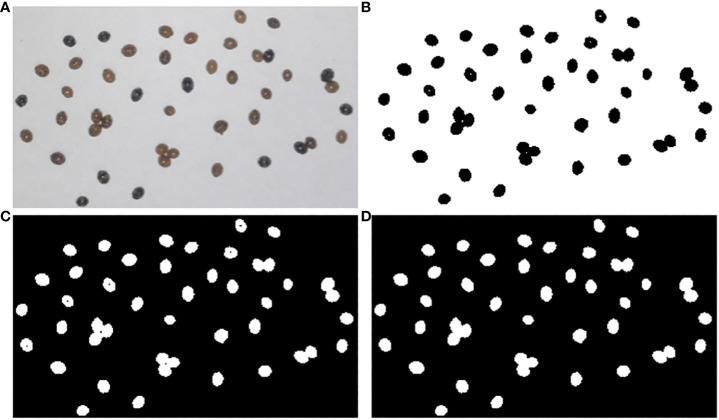
Image processing: **(A)** original image; **(B)** thresholding method; **(C)** thresholding inversion; **(D)** background fill.

**Figure 9:**
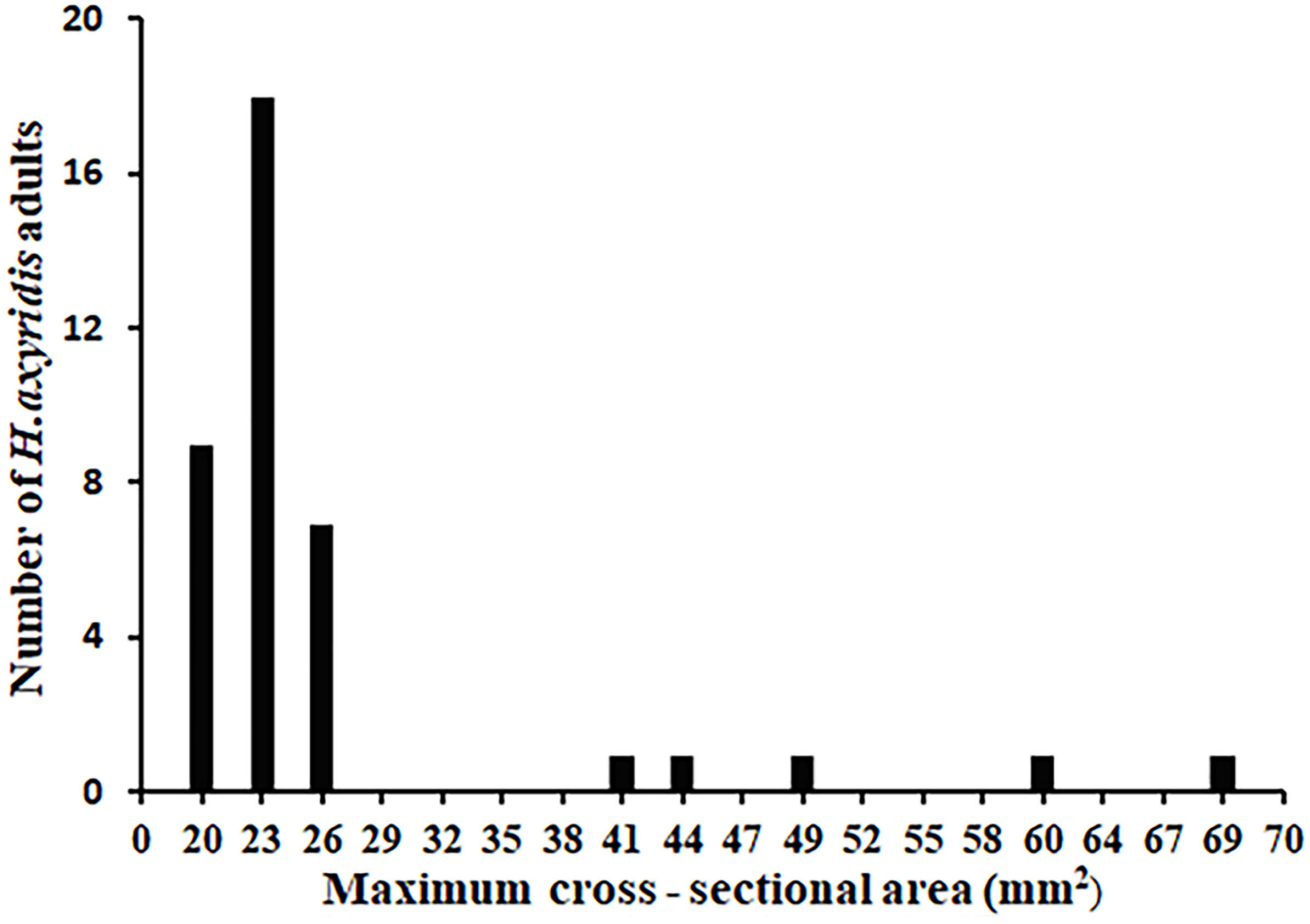
Maximum cross-sectional area statistics of *H. axyridis* adults.

The morphological characteristics of *H. axyridis* adults in the white paper box were photographed after the release. Subsequently, the images were processed and the number of *H. axyridis* adults was counted according to the procedure code in the Supporting Information. One of the boxes was selected and photographed. **Figure 8** illustrates the processing of the selected image. Using the maximum cross-linked area method, we performed a statistical analysis on the gathering behavior and obtained the cross-linked area of *H. axyridis* adult in the box. The results are presented in **Figure 9**.

The cross-sectional area of an individual *H. axyridis* adult ranged from 20 mm^2^ to 26 mm^2^, with a total of 34 *H. axyridis* adults. The total number of two connected *H. axyridis* adults was 3, with a combined cross-sectional area ranging from 41 mm^2^ to 49 mm^2^. Similarly, the total for three connected *H. axyridis* adults was 2, and the cross-sectional area ranges from 60 mm^2^ to 69 mm^2^. Based on the criterion of the range of cross-sectional area, it is possible to calculate the number of present *H. axyridis* adults.

During the release process, there were several *H. axyridis* adults in the white paper box. Even if there are severely connected *H. axyridis* adults in the white paper box, we can separate the severely attached *H. axyridis* adults by shaking the white paper box. Afterward, we can determine the number of them. To validate the maximum cross-sectional area method, we counted the number of *H. axyridis* adults in 100 white paper boxes, achieving an accuracy rate of 100%.

### 3.3 Performance of prototype

**Figure 10:**
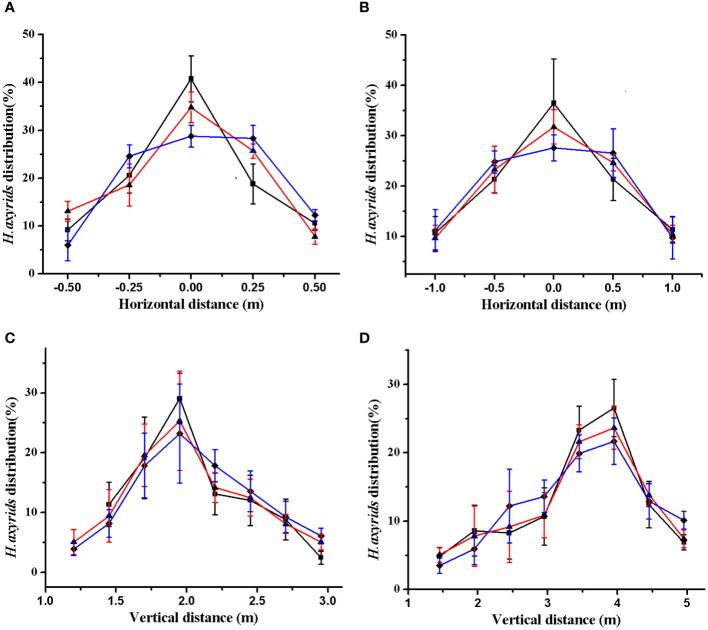
Distribution law. **(A)** horizontal distribution(29.5 m s^−1^); **(B)** horizontal distribution (38.3m s^−1^); **(C)** vertical distribution (29.5 m s^−1^); **(D)** horizontal distribution (38.3 m s^−1^). The black, red, and blue lines show the distribution of *H. axyridis* adults at the impeller rotating speeds of 4.2 rpm, 9.8 rpm, and 16.8 rpm, respectively.

To assess the distribution law of the prototype, we released the *H. axyridis* adults under the air velocity of 29.5 m/s and 38.3 m/s, with impeller rotating speeds of 4.2 rpm, 9.8 rpm, and 16.8 rpm. A schematic diagram of the release experiment can be seen in **Figure 6**. The distribution of *H. axyridis* adults is shown in **Figure 10**. As the impeller rotating speed increased, the distribution of *H. axyridis* adults in the horizontal direction became more uniform. However, the impeller speed had no significant effect on the distribution of *H. axyridis* adults in the vertical direction. This result may be attributed to the reduced time taken for the vanes to pass through the EA section as the impeller speed increases (see **Figure 3**). Before the space between the vanes can fill with *H. axyridis* adults, it’s already rotated to exit BC. The *H. axyridis* adults are then transported out by the airflow. The *H. axyridis* adults are then transported out by the airflow. At higher impeller rotating speeds, less space between the vanes can be filled. A higher number of releases is needed when the same number of *H. axyridis* adults. So Horizontal dispersion of *H. axyridis* adults becomes more even when they are released more frequently.

As for horizontal distribution, at an airflow velocity of 29.5m/s, at least 70% of the *H. axyridis* adults were distributed between −0.25 m and 0.25 m. At an airflow velocity of 38.3 m/s, the distribution of *H. axyridis* adults is between −0.5 m and 0.50 m. For vertical distribution at an airflow velocity of 29.5 m/s, at least 70% of *H. axyridis* adults are found between 1.75 m and 2.25 m. Similarly, at an airflow velocity of 38.3 m/s, at least 70% of *H. axyridis* adults can be found between 3.00 m and 4.5 m. The distribution of *H. axyridis* adults is influenced by variations in airflow velocity. Pezzi and coworkers (Pezzi et al., 2015) summarized the airflow distribution at the outlet speed of the 30m/s and 45m/s using a fan anemometer positioned at regular intervals. They found that with the 45 m/s setting the airflow was detectable at a distance of 6 m from the blower and had a width of 1.8 m, whereas with the 30 m/s setting this was reduced to 4.5 m with a width of 1.25 m. The results of this study show that the distribution area is larger at high airflow velocity compared to low airflow velocity, which is according to Pezzi’s Law.

Distribution uniformity, survival, and adhesion are the crucial parameters for evaluating the performance of the mechanical system. As shown in **Table 4**, the CV of horizontal and vertical distribution decreased with the increase of impeller rotating speed at certain airflow of the air channel. The CV of the horizontal direction was larger than that of the vertical direction, which indicated that the uniformity of the horizontal distribution was inferior to that of the vertical distribution. When the airflow velocities were 29.5 m/s and 38.3 m/s, the survival rates of the *H. axyridis* adults were 93.8% and 94.5% at 4.2 rpm, while the survival rates of *H. axyridis* adults were 78.6% and 78.7% at 16.8rpm. The low survival rate of *H. axyridis* adults at high-impeller rotating speed could be attributed to their injury upon contact with the mechanical wall. Martelli (Martelli et al., 2020) found that 88% of Orius laevigatus nymphs survived after release. In this study, the survival rate of *H. axyridis* adults was up to 94.5%. The difference in survival rate between the two studies may be due to the fact that Martelli released Orius laevigatus nymphs, while *H. axyridis* adults were released in this study. The streamlined shape of *H. axyridis* adults results in less damage when in contact with the machine wall.

**Table 4:** Coefficient of variation, survival, and adhesion of *H. axyridis* adults under different operating parameters.

Airflow speed	Impeller rotating	Horizontal	Vertical	Survival	Adhesion
(m s^−1^)	speed(rpm)	CV (%)	CV (%)	(%)	(%)
29.5	4.2	35.60 ± 3.39	24.03 ± 4.20	93.82 ± 2.79	4.63 ± 0.75
	9.8	33.37 ± 4.12	22.89 ± 3.48	89.06 ± 3.65	2.50 ± 1.49
	16.8	29.71 ± 2.56	18.84 ± 3.13	78.64 ± 4.04	2.87 ± 1.78
38.3	4.2	31.14 ± 2.49	23.27 ± 1.77	94.48 ± 3.09	4.13 ± 0.57
	9.8	28.31 ± 5.13	20.17 ± 2.11	88.19 ± 5.54	2.77 ± 1.15
	16.8	21.03 ± 3.62	14.78 ± 1.42	78.74 ± 3.41	3.50 ± 1.91

Based on the information presented in **Table 4**, a two-factor analysis of variance was conducted with survival rate as the dependent variable and impeller rotating speed and airflow velocity as the independent variables. The results indicate that airflow velocity did not have a significant impact on the survival rate of *H. axyridis* adults (*P* > 0.05). In contrast, impeller rotating speed had a significant effect on the survival rate of *H. axyridis* adults (*P*< 0.001). The survival rate of the *H. axyridis* adults decreased with the increase of impeller rotating speed. Considering the release survival rate and the area search ability of *H. axyridis* adults, a lower impeller rotating speed should be selected when releasing *H. axyridis* adults.

## 4 Conclusion

A mechanical system was developed for the release of *H. axyridis* adults, which combined centrifugal and pneumatic strategies. The impeller for transporting *H. axyridis* adults was designed based on the physical characteristics of the *H. axyridis* adults. A mobile phone and image processing method were employed to develop a technique for enumerating *H. axyridis* adults. The effects of different airflow velocities and impeller rotating speeds on the distribution and survival rate of *H. axyridis* adults were studied. The results showed that the impeller rotating speed had little effect on the vertical distribution of *H. axyridis* adults. At an airflow velocity of 29.5m/s, the distribution range of *H. axyridis* adults was 0.5 m × 0.5 m. At an airflow velocity of 38.3m/s, the distribution range of *H. axyridis* adults was 1.0 m × 1.5 m. The impeller rotating speed had a significant effect on the survival rate of *H. axyridis* adults. At low impeller rotating speed, the survival rate of the *H. axyridis* adults could reach 94.5%. This work presented equipment for the release of natural enemies, which will significantly increase the potential for the use of natural enemies to control pests and diseases in the field.

## Data availability statement

The original contributions presented in the study are included in the article/**Supplementary Material**. Further inquiries can be directed to the corresponding authors.

## Ethics statement

The manuscript presents research on animals that do not require ethical approval for their study.

## Author contributions

X-YD: Writing – original draft, Writing – review & editing. XT: Writing – original draft. JM: Writing – original draft. B-JQ: Writing – review & editing.
