# Supplementary material for: Design and evaluate the performance of a mechanical system for the release of Harmonia axyridis adults
Source: Front Plant Sci. 2024 Mar 5;15:1297182. doi: 10.3389/fpls.2024.1297182 (PMC10948399; doi:10.3389/fpls.2024.1297182)
Supplement: Supplementary file 1 [file DataSheet_1.pdf]

# Supporting Information

## Procedure code

```
clear all
close all
clc
P = 'E:\1\';
D = dir([P '*.jpg']);
for i = 1 : length(D)
    Image= imread([P D(i).name]);
    figure(1)
    imshow(Image)
    title('origin')
    Threshold=graythresh(Image);
    Image_BW=im2bw(Image,Threshold);
    figure(2)
    imshow(Image_BW);
    title('binaryzation')
    Reverse_Image_BW=~Image_BW;
    figure(3)
    imshow(Reverse_Image_BW);
    title('After optimization, the binary image is inverted')
    Filled_Image_BW=bwfill(Reverse_Image_BW,'holes');
    figure(4)
    imshow(Filled_Image_BW);
    title('The binary image has been filled with background color')
    [Label Number]=bwlabel(Filled_Image_BW,8);
    figure,imshow(Label);
    graindata=regionprops(Label,'basic');
        for j=1:Number
        if  graindata(j).Area>1180&&graindata(j).Area<1420
            N=1;
        else if  graindata(j).Area>1770&&graindata(j).Area<2130
            N=2;
        else if  graindata(j).Area>2360&&graindata(j).Area<2840
             N=3;
        else if  graindata(j).Area>2950&&graindata(j).Area<3550
              N=4;
         else
              N=0;

         end
             Number=Number+N;
            end
    disp(strcat('A',num2str(i),'=',num2str(Number)))
end
```
